# Effects of hemodynamic alterations and oxygen saturation on cerebral perfusion in congenital heart disease

**DOI:** 10.1038/s41390-024-03106-6

**Published:** 2024-03-04

**Authors:** Alexandra De Silvestro, Giancarlo Natalucci, Maria Feldmann, Cornelia Hagmann, Thi Dao Nguyen, Seline Coraj, Andras Jakab, Raimund Kottke, Beatrice Latal, Walter Knirsch, Ruth Tuura

**Affiliations:** 1 Pediatric Cardiology, Pediatric Heart Center, Department of Surgery, University Children’s Hospital Zurich, Zurich, Switzerland; 2 Center for MR-Research, University Children’s Hospital Zurich, Zurich, Switzerland; 3 Children’s Research Center, University Children’s Hospital Zurich, Zurich, Switzerland; 4 University of Zurich, Zurich, Switzerland; 5 Larsson-Rosenquist Foundation Center for Neurodevelopment, Growth and Nutrition of the Newborn, Department of Neonatology, University Hospital Zurich, Zurich, Switzerland; 6 Newborn Research Zurich, Department of Neonatology, University Hospital Zurich, Zurich, Switzerland; 7 Child Development Center, University Children’s Hospital Zurich, Zurich, Switzerland; 8 Department of Neonatology and Pediatric Intensive Care, University Children’s Hospital Zurich, Zurich, Switzerland; 9 Department of Diagnostic Imaging, University Children’s Hospital Zurich, Zurich, Switzerland

## Abstract

**Background:**

Patients with severe congenital heart disease (CHD) are at risk for neurodevelopmental impairment. An abnormal cerebral blood supply caused by the altered cardiac physiology may limit optimal brain development. The aim of this study was to evaluate the effect of a systemic-to-pulmonary shunt, aortic arch obstruction and arterial oxygen saturation on cerebral perfusion in patients with severe CHD.

**Methods:**

Patients with severe CHD requiring cardiac surgery within the first six weeks of life, who underwent pre- and/or postoperative brain magnetic resonance imaging (MRI), and healthy controls with one postnatal scan were included. Cerebral perfusion in deep and cortical gray matter was assessed by pseudocontinuous arterial spin labeling MRI.

**Results:**

We included 59 CHD and 23 healthy control scans. The presence of a systemic-to-pulmonary shunt was associated with decreased perfusion in cortical (*p* = 0.003), but not in deep gray matter (*p* = 0.031). No evidence for an effect of aortic arch obstruction and arterial oxygen saturation on cerebral perfusion was found. After adjusting for hemodynamic and oxygen saturation parameters, deep (*p* = 0.018) and cortical (*p* = 0.012) gray matter perfusion was increased in patients with CHD compared to controls.

**Conclusion:**

We detected regional differences in compensation to the cerebral steal effect in patients with severe CHD.

**Impact:**

- Patients with severe congenital heart disease (CHD) have altered postnatal brain hemodynamics.
- A systemic-to-pulmonary shunt was associated with decreased perfusion in cortical gray matter but preserved perfusion in deep gray matter, pointing towards regional differences in compensation to the cerebral steal effect.
- No effects of aortic arch obstruction and arterial oxygenation on cerebral perfusion were seen.
- Cerebral perfusion was increased in patients with CHD compared to healthy controls after adjusting for hemodynamic alterations and oxygen saturation.
- To improve neuroprotection and neurodevelopmental outcomes, it is important to increase our understanding of the factors influencing cerebral perfusion in neonates with severe CHD.

## Introduction

Infants with severe congenital heart disease (CHD) undergoing early cardiac surgery are at risk for perioperative brain injuries and neurodevelopmental impairment.^1,2^ Alterations in brain growth and maturation of this population are well described and have been observed to start in utero,^3–5^ but the underlying causes are still being investigated.

During the critical clinical course after birth, patients with severe CHD undergo early cardiac interventions including cardiopulmonary bypass surgery and treatment in the intensive care unit, putting them at risk for brain injuries.^6^ In addition to these treatment-related impacts, an abnormal cerebral blood supply caused by the pathophysiological effects of the cardiac disease may limit optimal brain development. Neuromonitoring techniques such as near-infrared spectroscopy (NIRS) and amplitude-integrated electroencephalography (aEEG) are increasingly applied to identify cerebral perfusion deficits which may otherwise escape the clinicians notice. However, to improve brain protection and neurodevelopmental outcomes, it is not only important to monitor, but also to increase knowledge about the factors influencing cerebral perfusion in these vulnerable patients with severe CHD.

Magnetic resonance imaging (MRI) methods such as pseudocontinuous arterial spin labeling (pCASL) and phase contrast imaging allow for objective quantification of cerebral perfusion.^7^ Since these techniques are non-invasive, using blood water as an endogenous contrast agent in pCASL or by measuring blood flow in phase contrast imaging, they are applicable in vulnerable patient cohorts such as patients with severe CHD.^7^

In a healthy brain, cerebrovascular autoregulation provides a stable cerebral blood flow despite changes in cerebral perfusion pressure,^8^ regulated through changes in arteriolar diameter.^9^ In fetuses with severe CHD, several ultrasound studies described a so-called fetal brain-sparing effect, preserving blood flow via cerebral vasodilation by a decreased cerebral-to-placental resistance ratio.^10^ In MRI perfusion analyses after birth, one study found preoperative cerebral hypoperfusion in patients with single ventricle CHD,^11^ whereas other studies described no cerebral perfusion deficits in CHD patients as compared to healthy controls.^12,13^ However, given the variety of included CHD diagnoses in those studies, a more detailed investigation of cerebral perfusion and potential influencing factors is needed.

In the treatment of patients with severe CHD, systemic-to-pulmonary shunts (SPS) are often required to improve or obtain pulmonary perfusion. SPS include for example preserving patency of the arterial duct by prostaglandin E2, stenting of the arterial duct, or surgically created modified Blalock-Taussig (mBT) shunts. A SPS can potentially lead to an unfavorable imbalance of systemic to pulmonary perfusion,^14^ known as the shunt steal effect, resulting in a decrease in diastolic flow or even a run-off of blood from the arteries supplying the brain and other systemic organs into the pulmonary circulation.^15^ Additional pathophysiological factors influencing cerebral perfusion in patients with severe CHD are the presence of an aortic arch obstruction^11^ and decreased arterial oxygen saturation.^16^ Aortic arch obstruction may increase cerebral perfusion by restricting blood flow to the descending aorta.^11^ On the other hand, decreased arterial oxygen saturation may increase cerebral perfusion through hypoxia-induced cerebral vasodilation.^16^ MRI studies investigating the effects of these parameters in infants with CHD are scarce.

We aimed to evaluate the effects of the SPS, aortic arch obstruction and arterial oxygen saturation on cerebral perfusion in CHD infants using pCASL MRI. Furthermore, we investigated differences in cerebral perfusion between CHD patients and healthy controls. We hypothesized that 1) the presence of a SPS, aortic arch obstruction and arterial oxygen saturation in CHD patients affect cerebral perfusion and 2) cerebral perfusion is impaired in CHD patients as compared to healthy controls.

## Methods

### Study design and patient population

This analysis was conducted as a secondary evaluation of two cohort studies. In one study, patients with severe CHD as defined by undergoing cardiac surgery within the first six weeks of life were consecutively recruited from 2013 to 2020 at the University Children’s Hospital Zurich, and a healthy control group was recruited in parallel. Further healthy control subjects were included from a second study with ongoing recruitment since 2017, which utilized the same MRI protocol and scanner. All subjects were term born. Patients with genetic diagnoses were excluded. Written informed consent was obtained from all participant’s parents. Both studies were approved by the ethical committee of Kanton Zürich, Switzerland (Ref.No. StV 19/04 and BASEC 2019-01993).

### Brain MRI

Brain MRI scans were performed before and after cardiac surgery in CHD patients, and at one postnatal time point in healthy controls. Scans were conducted in natural sleep using the feed-and-sleep method, and noise protection was provided by earplugs and Minimuffs. Patients were hemodynamically stable and normotensive at the time of the scan and monitored with pulse oximetry. Scans were performed on a 3T GE MR750 MRI scanner using an 8-channel head coil. The scanning protocol has been described previously^17^ and included T2-weighted fast-spin-echo sequences in three planes, a 3D T1-weighted sequence, diffusion tensor imaging, single voxel short TE MR spectroscopy of the basal ganglia and white matter^18^ and pCASL sequences. The resulting images were analyzed by a neuroradiologist for abnormalities (R.K.). Patients with pathological lesions with a possible impact on cerebral perfusion, defined as intraventricular hemorrhage with extension into the lateral ventricles (grade ≥2), ischemic lesions and hypoxic-ischemic encephalopathy, were excluded. Scans with non-expansive/small birth-related subdural hematomas and punctuate white matter lesions were included.

### Cerebral perfusion assessment

**Fig. 1:**
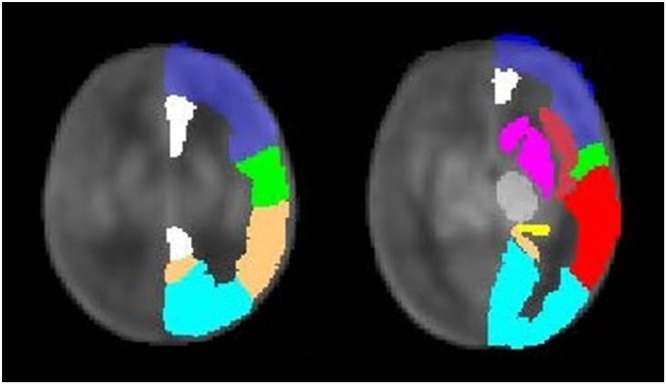
Regional Automated Anatomical Labeling masks superimposed on the study perfusion template. Regions of interest are depicted as follows, dark blue: frontal region, green: paracentral region, copper: parietal region, light blue: occipital lobe, white: cingulate gyrus, dark-red: insula, red: temporal lobe, yellow: hippocampus, gray: thalamus, magenta: basal ganglia.

Cerebral perfusion images were acquired with a background-suppressed pCASL sequence using a 3-dimensional (3D) stack of spirals readout with a post-labeling delay of 2.025 seconds. Imaging parameters were: field of view 180 mm, slice thickness 4 mm, repetition time 4679 ms, echo time 10.64 ms, number of averages 3, reconstruction matrix 128 × 128. Quantitative pCASL perfusion maps were reconstructed with the vendor-provided perfusion quantification software. The perfusion images were normalized to a study-specific, neonatal perfusion template using the linear registration tool in FSL-FLIRT.^19^ Quality check of pCASL sequences was done visually. Sequences with motion and/or spiral artefacts or incomplete depictions of the cerebral regions of interest were excluded. Regional perfusion was assessed using the Automated Anatomical Labeling (AAL) atlas masks^20^ (Fig. 1) in deep gray matter (dGM: basal ganglia, thalamus) and in cortical gray matter (cGM: frontal, parietal, paracentral region, temporal and occipital lobe, cingulate gyrus, hippocampus, insula), in ml/min/100 g brain tissue. White matter perfusion was not considered because of the low signal-to-noise ratio. Regional perfusion values were calculated as the average of both brain hemispheres. T1-correction for the effect of hematocrit on perfusion values was applied.^21^

Cerebral oxygen delivery (CDO_2_, in ml/min/100 g brain tissue) was calculated by applying the formula by Rudolph et al.^22^ used in previous CHD studies,^13,23–26^ including arterial oxygen saturation (SaO_2_), hemoglobin concentration (Hgb), Hüfner’s constant (1.36) and cerebral blood flow (CBF, equivalent to cerebral perfusion as used in this study): CDO_2_ = SaO_2_ x Hgb x 1.36 x CBF.

### Clinical parameters

The presence of a shunt between the systemic and pulmonary circulation was evaluated by a pediatric cardiologist (W.K.) and categorized into SPS and pulmonary-to-systemic shunts (PSS) as defined by the main blood flow direction in the echocardiography report. Shunt types included anatomical shunts such as patent ductus arteriosus (PDA, open due to systemic prostaglandin E2 infusion after birth) or common arterial trunk, as well as interventional/surgical shunts such as stented PDA, mBT shunt (connecting the right subclavian artery to the right pulmonary artery) and central aortopulmonary shunt (connecting the ascending aorta and the central pulmonary artery/bifurcation).

The effect of the shunt was analyzed in a subset of patients with and without SPS, excluding patients with PSS and common arterial trunk. One patient with PDA and interrupted aortic arch Type A was assigned to the group without SPS due to the absence of a potential cerebral steal effect. Relative shunt-size was defined as the smallest shunt diameter determined by echocardiography (mm) to body weight (kg). Aortic arch obstruction included hypoplasia and coarctation of the aorta, as well as interrupted aortic arch. The presence of a shunt and aortic arch obstruction was defined at the time point of the MRI scan. Arterial oxygen saturation was measured during the scans using a transcutaneous probe (SpO_2_). The hematocrit and hemoglobin values for the patients with CHD were taken from clinical blood analyses, and values for healthy controls were defined using mean age-adapted normative values.^27^

### Statistics

Statistical analyses were conducted using R (version 4.2.2, The R Software Foundation for Statistical Computing, Vienna, Austria). Continuous data were assessed for normality by visual inspection of histograms and Q-Q plots and reported as mean and standard deviation, or median and interquartile range, respectively. Categorical data were presented as numbers and percentages. A few cases lacked documentation of oxygen saturation at scan (7 healthy controls and 7 postoperative patients with CHD, all with saturations of 99-100% before scan), and missing values were replaced with the mean value of the healthy controls.

Four statistical models were applied. The effects of postmenstrual age, SPS, aortic arch obstruction and arterial oxygen saturation on cerebral perfusion in patients with severe CHD were evaluated using a linear mixed effects model (Model 1). The effect of shunt size in CHD patients with PDA was assessed using a linear model (Model 2, all patients in this group were scanned only once). Differences between patients with CHD and healthy controls were assessed using two linear mixed-effects models (Models 3 and 4).

Model 1:

cerebral perfusion = intercept + β1 * postmenstrual age (weeks) + β2 * shunt (yes/no) + β3 * aortic arch obstruction (yes/no) + β4 * oxygen saturation (%) + b0s

Model 2:

cerebral perfusion = intercept + β1 * postmenstrual age (weeks) + β2 * relative shunt size (mm/kg)

Model 3

cerebral perfusion = intercept + β1 * postmenstrual age (weeks) + β2 * study group (CHD/controls) + b0s

Model 4

cerebral perfusion = intercept + β1 * postmenstrual age (weeks) + β2 * shunt (yes/no) + β3 * aortic arch obstruction (yes/no) + β4 * oxygen saturation (%) + β5 * study group (CHD/controls) + b0s

β’s denote the fixed effects slopes, and b0s denotes the random intercept for subjects scanned both pre- and postoperatively.

Covariates of all models were included for theoretical criteria, based on literature reports of their effect on cerebral perfusion.^11,15,16^ Models 1 and 2 were applied in patients with CHD only, whereas models 3 and 4 included patients and healthy controls. In healthy controls, shunt and aortic arch obstruction were absent by definition. The models were applied for dGM and cGM perfusion as primary analysis. Alpha significance level was set to 0.025, using Bonferroni correction for testing in two gray matter regions (dGM and cGM). For a secondary analysis, regional perfusion was assessed in 10 subregions as listed above, using Benjamini-Hochberg adjusted *p*-values with a false discovery rate of 0.025 to account for multiple testing. For an additional analysis of CDO_2_, covariates of Model 3 and Model 4 were applied, excluding the covariate “oxygen saturation”. Interactions between covariates were tested in all models and omitted after a negative significance check.

## Results

**Fig. 2:**
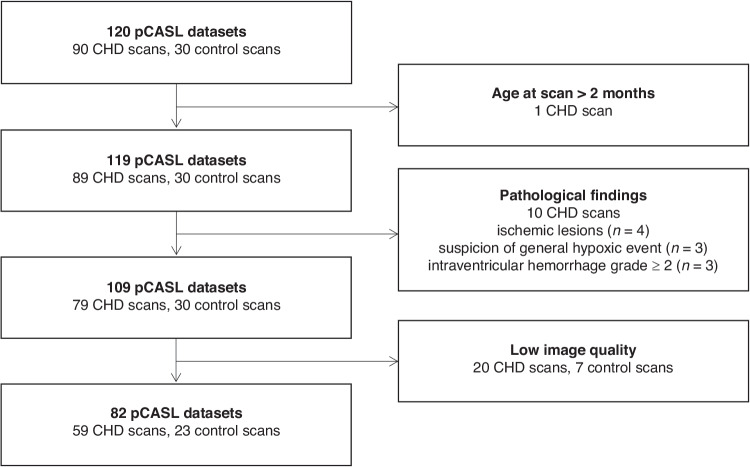
Flow diagram of pCASL data inclusion. *CHD* congenital heart disease, *pCASL* pseudocontinuous arterial spin labeling.

A total of 120 pCASL datasets were available, including 90 of patients with CHD and 30 of healthy controls. ASL data was excluded due to advanced age at scan (>2 months), low image quality or pathological findings (Fig. 2). The final dataset included 82 cerebral MRI scans, consisting of 59 CHD (27 pre-, 32 postoperative; mean PMA 41.9 weeks, range 37.9-47.1) and 23 control scans (mean PMA 41.6 weeks, range 38.2-46.1). Nine patients had both pre- and postoperative scans, resulting in a study group of 73 infants (50 patients and 23 healthy controls). The most frequent type of CHD was d-Transposition of the Great Arteries (d-TGA, *n* = 23). Patient characteristics are presented in Table 1. Gestational age, body weight and head circumference at birth, as well as age at scan (both PMA and chronological age) were similar for infants with CHD and healthy controls.

**Table 1:** Patient characteristics.

	CHD patients	Healthy controls	*p* value
**Subjects**			
*n*	50	23	
Sex, female (%)	13 (26)	13 (57)	0.023*
Gestational age at birth, weeks (mean (SD))	39.4 (1.3)	39.2 (1.4)	0.41
Body weight at birth, gram (mean (SD))	3330 (500)	3220 (360)	0.34
Body length at birth, cm (mean (SD))	49.1 (3.9)	49.1 (1.8)	0.99
Head circumference at birth, cm (mean (SD))	34.7 (1.5)	34.6 (1.1)	0.65
Type of CHD (%)^a^			-
Transposition of Great Arteries	23 (46)		
Aortic Arch Anomaly	8 (16)		
Single Ventricle	8 (16)		
Right Ventricular Outflow Tract Obstruction	4 (8)		
Total Anomalous Pulmonary Venous Drainage	4 (8)		
Other	3 (6)		
**pCASL datasets**			
*n*	59	23	
Postmenstrual age at scan, weeks (mean (SD))	41.9 (2.1)	41.6 (1.8)	0.63
Chronological age at scan, days (mean (SD))	16.3 (11.1)	16.7 (7.7)	0.86
Shunt at scan (%)	28 (47)		–
Systemic-to-pulmonary shunt	24 (41)		
Pulmonary-to-systemic shunt	3 (5)		
Common arterial trunk	1 (2)		
Aortic arch obstruction at scan (%)	8 (14)		–
O_2_ Saturation at scan, % transcut. (mean (SD))	89.4 (6.4)	94.0 (1.7)	0.001*
Hemoglobin, g/dl (mean(SD))	14.0 (2.5)	15.0 (1.6)	0.10
Hematocrit, % (mean (SD))	40.9 (6.8)	45.7 (5.3)	0.003*

*pCASL* pseudocontinuous arterial spin labeling, *CHD* congenital heart disease, *O*_*2*_ oxygen, *transcut*. transcutaneous.

^a^Transposition of Great Arteries including d-Transposition of Great Arteries (d-TGA) (*n* = 20) and Double Outlet Right Ventricle (DORV) TGA type (*n* = 3).

Aortic Arch Anomaly including Coarctation of the Aorta (*n* = 2), Hypoplastic (*n* = 4) and Interrupted Aortic Arch (*n* = 2).

Single Ventricle including Hypoplastic Left Heart Syndrome (*n* = 2), Hypoplastic Left Heart Complex (*n* = 1), Double Inlet Left Ventricle (*n* = 2), Pulmonary Atresia with intact Ventricular Septum (*n* = 1); Tricuspid Atresia (*n* = 1), Left Atrial Isomerism (*n* = 1).

Right Ventricular Outflow Tract Obstruction including Pulmonary Atresia with VSD (*n* = 2), Double Outlet Right Ventricle (DORV) Fallot type (*n* = 2).

Others including Ventricular Septal Defect (*n* = 1), Common Arterial Trunk (*n* = 1), congenital corrected TGA (L-TGA) (*n* = 1).

*p < Bonferroni-corrected alpha significance level 0.025.

### Effects of hemodynamic alterations and oxygen saturation on cerebral perfusion in patients with CHD

The effects of hemodynamic alterations and oxygen saturation on cerebral perfusion were evaluated in a total of 55 CHD scans, excluding scans with pulmonary-to-systemic shunt (*n* = 3) and common arterial trunk (*n* = 1). SPS was present in 24 (44%) scans, including 19 scans with preoperative PDA (CHD types: d-TGA (*n* = 13), aortic arch anomaly (*n* = 3), right ventricular outflow tract obstruction (RVOTO, *n* = 3), congenital corrected TGA (L-TGA, *n* = 1)), 3 scans with mBT-shunt (single ventricle CHD, *n* = 3) and one scan with central aortopulmonary shunt (RVOTO, *n* = 1). Aortic arch obstruction was present in 7 preoperative scans (CHD types: aortic arch anomaly (*n* = 4), d-TGA (*n* = 2), L-TGA (*n* = 1)). Six of the seven patients with aortic arch obstruction additionally had a SPS at scan. Mean (SD) arterial oxygen saturation at the timepoint of scan was 90 (6.5) %.

The results of Model 1 are presented in Table 2. In patients with severe CHD, the presence of a SPS was associated with decreased perfusion in cGM (*p* = 0.003). No evidence for an effect of SPS on dGM was found (*p* = 0.031). Perfusion in both dGM and cGM increased with postmenstrual age. A plot visualizing cerebral perfusion for SPS vs. non-SPS patients is provided in the Supplement, Figure I.

**Table 2:** Results of Model 1. Effect of postmenstrual age, systemic-to-pulmonary shunt, aortic arch obstruction and oxygen saturation on cerebral perfusion in deep and cortical gray matter.

	Beta	95% CI	*p* value
**Perfusion in deep Gray Matter**			
Intercept	−9.65	−51.52–31.98	0.66
Postmenstrual age, weeks	1.34	0.53–2.19	0.003*
Systemic-to-pulmonary shunt: yes	−4.04	−8.23–−0.24	0.031
Aortic arch obstruction: yes	5.06	−0.44–11.05	0.04
O_2_ Saturation, %	−0.20	−0.45–0.05	0.13
**Perfusion in cortical Gray Matter**			
Intercept	1.71	−21.94–25.57	0.89
Postmenstrual age, weeks	0.76	0.30–1.22	0.003*
Systemic-to-pulmonary shunt: yes	−3.36	−5.47–−1.30	0.003*
Aortic arch obstruction: yes	0.82	−2.00–3.62	0.58
O_2_ Saturation, %	−0.12	−0.27–0.03	0.13

*CI* confidence interval, *O*_*2*_ oxygen.

*p < Bonferroni-corrected alpha significance level 0.025.

#### Subregion analysis

Analyzing gray matter subregions, the SPS was significantly associated with decreased perfusion in 4 of the 8 cortical subregions: in frontal (*p* = 0.014), parietal (*p* = 0.016), paracentral (*p* = 0.015) region, as well as in cingulate gyrus (*p* = 0.014). No evidence for a SPS effect in dGM subregions was found. Furthermore, we observed no evidence for an effect of aortic arch obstruction or oxygen saturation in any subregion.

#### Cerebral oxygen delivery

No evidence for an effect of SPS or aortic arch obstruction, nor age-dependence was found on both dGM and cGM CDO_2_ (see Supplementary Table I and Figure I).

#### Relative shunt size

**Fig. 3:**
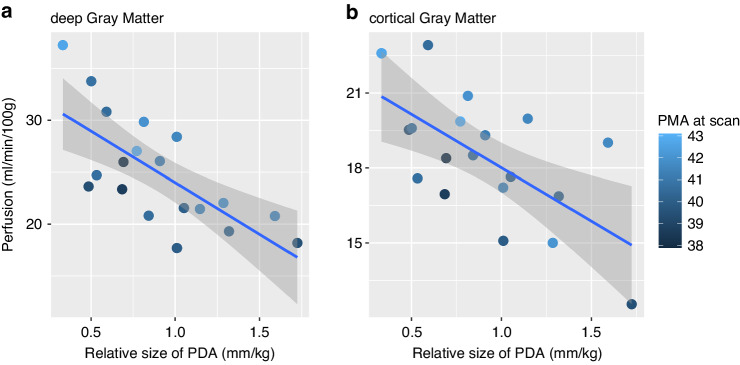
Association of relative size of patent ductus arteriosus and cerebral perfusion. Left panel **a**: Association between shunt size and perfusion in deep gray matter. Right panel **b**: Association between shunt size and perfusion in cortical gray matter. PDA patent ductus arteriosus, PMA postmenstrual age (weeks). Note: Plots were not adjusted for postmenstrual age (but visualized by color coding), presence of aortic arch obstruction and arterial oxygen saturation.

The effect of relative shunt size on cerebral perfusion was evaluated in a subgroup of CHD patients with PDA type of SPS (*n* = 19), correcting for PMA at scan (Model 2). A significant negative association of relative shunt size and cerebral perfusion in both dGM and cGM was found, see Fig. 3 (*p* ≤ 0.001 in dGM, *p* = 0.001 in cGM).

### Differences between patients with CHD and healthy controls

Using the simple Model 3 adjusting for PMA only, no evidence for differences in cerebral perfusion between patients and controls was found both in dGM and cGM.

After additionally adjusting for presence of SPS, aortic arch obstruction and oxygen saturation in Model 4, we found significantly increased perfusion in patients with CHD as compared to controls in dGM (*p* = 0.018) and in cGM (*p* = 0.012). Results are shown in Tables 3 and 4. Model 4 performed better than Model 3 in prediction of perfusion in dGM (conditional R^2^ of Model 3 = 0.31, Model 4 = 0.52), as well as in cGM (conditional R^2^ of Model 3 = 0.28, Model 4 = 0.35).

**Table 3:** Results of Model 3. Effect of postmenstrual age and study group on cerebral perfusion in deep and cortical gray matter.

	Beta	95% CI	p value
**Perfusion in deep Gray Matter**			
Intercept	−31.77	−55.18–−8.35	0.010*
Postmenstrual age, weeks	1.35	0.79–1.91	<0.001*
*Study group: CHD*	*2.84*	*0.37–5.30*	*0.028*
**Perfusion in cortical Gray Matter**			
Intercept	−20.53	−35.11–−5.96	0.008*
Postmenstrual age, weeks	0.97	0.62–1.31	<0.001*
*Study group: CHD*	*1.14*	−*0.39–2.67*	*0.15*

*CHD* congenital heart disease, *CI* confidence interval.

*p < Bonferroni-corrected alpha significance level 0.025.

**Table 4:** Results of Model 4. Effect of postmenstrual age, study group, systemic-to-pulmonary shunt, aortic arch obstruction and oxygen saturation on cerebral perfusion in deep and cortical gray matter.

	Beta	95% CI	p value
**Perfusion in deep Gray Matter**			
Intercept	−8.47	−41.60–24.88	0.63
Postmenstrual age, weeks	1.16	0.53 – 1.81	<0.001*
Systemic-to-pulmonary shunt: yes	−3.84	−7.50–−0.40	0.024*
Aortic arch obstruction: yes	4.23	−0.67–9.55	0.06
O_2_ Saturation, %	−0.17	−0.40–0.07	0.16
*Study group: CHD*	*3.57*	*0.75–6.37*	0.018*
**Perfusion in cortical Gray Matter**			
Intercept	1.02	−18.64–20.69	0.92
Postmenstrual age, weeks	0.70	0.33–1.07	<0.001*
Systemic-to-pulmonary shunt: yes	−3.37	−5.31–−1.44	0.001*
Aortic arch obstruction: yes	0.68	−1.95–3.32	0.62
O_2_ Saturation, %	−0.11	−0.25–0.03	0.12
*Study group: CHD*	*2.29*	*0.60–3.98*	0.012*

*CHD* congenital heart disease, *CI* confidence interval, *O*_*2*_ oxygen.

*p < Bonferroni-corrected alpha significance level 0.025.

Patients with pulmonary-to-systemic shunt (*n* = 3) or common arterial trunk (*n* = 1) were excluded from analysis in Model 4.

#### Subregion analysis

Using model 4 for subanalysis, regional perfusion was increased in patients with CHD versus controls in basal ganglia (*p* = 0.019), paracentral region (*p* = 0.019), cingulate gyrus (*p* = 0.021), insula (*p* = 0.019) and hippocampus (*p* = 0.018).

#### Cerebral oxygen delivery

We found no evidence for differences in dGM and cGM oxygen delivery between patients with CHD and controls both in the simple (Model 3) and complex model (Model 4).

## Discussion

In this study which examined the effects of hemodynamic alterations and oxygen saturation on cerebral perfusion in infants with CHD, we found the presence of a SPS to be negatively associated with perfusion in the cGM, but not in the dGM. Furthermore, infants with CHD had increased cerebral perfusion in cGM when compared to healthy controls, after controlling for SPS, aortic arch obstruction and oxygen saturation.

### Effect of systemic-to-pulmonary shunt

The cerebral steal effect caused by a SPS is well-known and has been evaluated by Doppler ultrasound, presenting as low end-diastolic velocities with increased resistance index^28^ and pulsatility index^29^ in cerebral arteries. To our knowledge, this is the first study evaluating the association of this effect with regional cerebral perfusion using pCASL MRI data.

We found a strong inverse correlation of relative shunt size and cerebral perfusion in both dGM and cGM. This finding is in line with a study describing an inverse correlation of aortopulmonary collateral flow and whole-brain perfusion at 0.5 years in univentricular patients prior to Glenn procedure,^30^ using cardiac phase-contrast MRI data.

Whereas we found perfusion in dGM to be strongly dependent on relative shunt size, no evidence for a significant impact of the presence of SPS (vs. absence of SPS) on dGM perfusion was detected. In contrast, the presence of a SPS significantly decreased cGM perfusion. We conclude that in patients with a SPS who have low diastolic blood flow in cerebral arteries, perfusion seems to be prioritized in dGM with a lack of adequate compensation mechanisms in cGM areas, in particular in frontoparietal regions. The frontoparietal region is known to be the most perfused cortical region in neonates, and frontoparietal perfusion increases further after birth.^31,32^ Frontoparietal networks also support cognitive functions, like executive functions, which are known to be affected in children and adults with CHD.^33^ An abnormal frontoparietal perfusion in infancy due to the presence of a SPS may therefore increase the risk for subsequent neurodevelopmental or cognitive impairment, but further studies would be needed to investigate the link between SPS, perioperative perfusion changes and neurodevelopmental outcome in infants with CHD.

Although scans with the presence of SPS were conducted more often pre- than postoperatively, no evidence for a significant interaction between SPS and postmenstrual age was found.

### Effect of aortic arch obstruction and arterial oxygen saturation

Aortic arch obstruction and oxygen saturation were not significantly associated with cerebral perfusion in both dGM and cGM. This lack of a significant effect of aortic arch obstruction may have been caused by different degrees of aortic arch obstructions, which have not been assessed in detail for this study. Furthermore, the effects underlying the responses of hypoxia on cerebral perfusion are highly complex and the cerebral vasodilation has been reported in acute hypoxia, whereas the effect of more chronic hypoxia, especially in patients with CHD, is not yet well described.^16^ In contrast to our findings, Nagaraj et al.^11^ reported increased perfusion in patients with aortic arch obstruction as compared to those without, as well as lower regional perfusion in cyanotic than acyanotic types of CHD. However, they investigated these effects separately and did not adjust for the presence of SPS, which may have resulted in the divergent findings. In the present study, both aortic arch obstruction and oxygen saturation appear to have less impact on perfusion than the presence of a SPS.

### Differences between infants with CHD and healthy controls

Whereas we found no significant differences in cerebral perfusion between patients with CHD and controls only adjusting for PMA, we found increased gray matter perfusion in patients with CHD after adjusting for the presence of SPS, aortic arch obstruction and oxygen saturation. The complex statistical model was a better fit for the prediction of cerebral perfusion than the simple model in both gray matter regions. This confirms that hemodynamic and oxygen saturation parameters explain part of the variance in cerebral perfusion of patients with severe CHD and need to be taken into account when investigating their brain development.

So far, only few studies have evaluated differences in cerebral perfusion between patients with CHD and healthy controls. Similar to our findings, previous studies didn’t find evidence for significant differences in global and regional perfusion between patients with CHD and healthy controls, adjusting for postmenstrual age only, using both pCASL^11^ and phase-contrast^23^ MRI sequences. After taking SPS, aortic arch obstruction and oxygen saturation into account, we found increased gray matter perfusion in infants with CHD vs. controls. For patients with altered hemodynamic and oxygen saturation parameters, this implies a compensatory mechanism to provide optimal cerebral perfusion and oxygen supply. It also suggests that a brain-sparing effect may persist after birth. This brain-sparing effect has been described in fetuses with severe CHD, consisting of a relative decrease in cerebrovascular resistance to placental artery resistance to increase cerebral perfusion.^10^

On the other hand, the CHD group also consisted of patients with surgically corrected CHD. Gray matter perfusion was increased in patients with CHD independent of the presence of a SPS, aortic arch obstruction and oxygen saturation. This relative hyperperfusion may be explained by either an overcompensated cerebral perfusion after normalization of hemodynamic parameters and oxygen saturation, or may be a result of a (temporary) increase in cardiac output, e.g. by medication. The effect of medication on cerebral perfusion has not been investigated in this study due to the limited sample size. Preoperative administration of prostaglandin was very common in this study population, and postoperatively, diuretics, antihypertensive drugs, analgesics, anticoagulants and antibiotics were used, potentially impacting cerebral autoregulation. The effect of these drugs on cerebral perfusion in infants with CHD has not yet been investigated in the literature. None of the included patients were under catecholamines or milrinone (phosphodiesterase III inhibitor) at the time of scanning.

### Regional differences in cerebral perfusion

We found different regional perfusion patterns in gray matter, with cGM perfusion varying with hemodynamic influences (particularly the presence of a SPS), whereas dGM perfusion was more consistent.

In healthy infants, (whole-brain) cerebral perfusion increases rapidly with age and is thought to reflect areas undergoing development, including synaptogenesis and myelination. At newborn age, cGM perfusion is lower than dGM perfusion. Within the first months of life, relative cGM perfusion increases, putatively as a result of increased sensory input after birth. On the other hand, dGM perfusion stays more constant and relative dGM perfusion decreases.^34–37^

Therefore, cGM perfusion appears to be subject to greater changes in the neonatal period and may be more vulnerable to hemodynamic influences than dGM. Furthermore, gray matter maturation has been shown to be delayed in patients with severe CHD, with lower pre-^38^ and postnatal^39^ volumes as compared to healthy controls, and differences were accentuated in cGM.^38^ Brain immaturity may therefore additionally play a role in perfusion regulation, as it has already been shown to impact the cerebrovascular autoregulatory range.^40,41^ Alternatively, the relationship between brain immaturity and perfusion may be bidirectional, since in adults with arterial disease a reduced perfusion has also been related to a greater progression of brain atrophy over time.^42^ However, within the CHD population the directionality of the link between cGM maturation, brain volumes and altered perfusion remains unclear.

### Effects of age and sex

We confirmed that as in healthy infants, cerebral perfusion in patients with CHD increases with age, similar to results reported in a previous study.^11^ We found no evidence for a difference in this age-dependent perfusion increase in CHD with vs. without SPS, or in patients vs. healthy controls. Sex was included as a covariate in all of our statistical models evaluating cerebral perfusion for exploratory reasons, given the known sex differences in perfusion which emerge later in childhood and adolescence^43^ and persist into adulthood.^44,45^ However, no evidence for an effect of sex on cerebral perfusion was found, and the covariate was subsequently omitted due to the limited sample size. No study assessing sex-differences in cerebral perfusion of CHD or healthy neonates was found for comparison.

### Impact of cerebral perfusion on neurodevelopment

The impact of cerebral perfusion alterations in infants with CHD on neurodevelopmental outcomes has not yet been established.^46^ The investigation of direct associations of cerebral perfusion and neurodevelopmental outcome in these patients may be challenging as the influencing factors alter with time, e.g. by changes of cardiac structure and function with surgical and medical treatment, and measurement of cerebral perfusion using MRI reflects the current hemodynamic situation only. In this study, we found alterations in cerebral perfusion in patients with CHD, whereas CDO_2_ was consistent, irrespective of hemodynamic parameters, as well as between CHD and healthy infants. Compensatory mechanisms such as hyperperfusion in patients with CHD (as compared to controls), or an increase in hemoglobin in patients with SPS (as compared to patients without SPS) may therefore lead to adequate cerebral oxygen supply. It remains unclear if the supply of other nutrients (e.g. glucose) is adequately compensated in patients with SPS. In a study evaluating preterm patients, a longer duration of open PDA (with potential cerebral steal effect) has been associated with decreased cerebellar volumes.^47^ Further studies are needed to investigate the interplay between cerebral perfusion and brain maturation in patients with CHD. Our findings suggest that the frontoparietal region in particular is vulnerable to low blood supply in patients with SPS, and studies focusing on the maturation of this region would be of value.

### Limitations

This study has several limitations. A well-known challenge in CHD research is the heterogeneity of diseases. Using a multivariate model, we tried to overcome this difficulty and were able to gain knowledge of pathophysiologic factors influencing cerebral perfusion. Nevertheless, we were unable to investigate the effect of a pulmonary-to-systemic shunt on cerebral perfusion, or more general impact factors such as cardiac output, blood pressure or medication, due to the limited sample size and exploratory design of the study. Arterial oxygen saturation was measured via an MRI compatible transcutaneous probe, which has been shown to underestimate true oxygen saturation (mean value in healthy controls was 94%). Since oxygen saturation was measured using the same probe in all patients, the impact of this measurement error is thought to be minimal. However, measurement of the partial pressure of oxygen (PaO_2_) would have been beneficial.

The CHD group showed a wider distribution of ages at scan, but we found no evidence for significant groupwise differences in gestational or postnatal age at scan, nor a significant interaction of PMA and study group in the models.

Pre-defined AAL masks were applied for regional perfusion evaluation in gray matter. Border zone regions between cerebral vascular territories are most vulnerable to hypoperfusion and were not specifically evaluated in this study.

The association of Doppler ultrasound parameters and MRI perfusion variables would be of high clinical interest, because ultrasound is more accessible and suitable as a bed-side tool for clinical monitoring of the cerebral steal effect.^28^ Unfortunately, the association of routinely generated Doppler ultrasound and pCASL perfusion data could not be adequately analyzed in this study, due to the differing time points of examination (median 5, IQR 4–7 days between ultrasound and MRI). Cerebral perfusion between these time points may vary based on time-dependent changes in oxygen saturation and medication, as well as with age.

## Conclusion

In conclusion, we found an impact of pathophysiologic effects on cerebral perfusion in infants with severe CHD. Specifically, the presence of a SPS was associated with relative hypoperfusion in cGM but not in dGM, which points towards a regional difference in compensatory mechanisms to the cerebral steal effect. Moreover, after adjusting for SPS, aortic arch obstruction and oxygen saturation, gray matter perfusion was increased in patients with CHD as compared to healthy controls. Further investigations are needed to study the underlying causes of altered cerebral perfusion in this population and its interplay with brain maturation.

## Supplementary information

supplementary information

## Data Availability

The datasets analyzed during the current study are not publicly available due to ethical restrictions of patient data but are available from the corresponding author on reasonable request.
