# Supplementary material for: Effects of hemodynamic alterations and oxygen saturation on cerebral perfusion in congenital heart disease
Source: Pediatr Res. 2024 Mar 4;96(4):990–8. doi: 10.1038/s41390-024-03106-6 (PMC11502515; doi:10.1038/s41390-024-03106-6)
Supplement: Supplementary file 1 — supplementary information [file 41390_2024_3106_MOESM1_ESM.pdf]

Supplementary Material

Table I. Effect of postmenstrual age, systemic-to-pulmonary shunt and aortic arch obstruction on cerebral oxygen delivery in deep and cortical gray matter.

	Beta	95% CI	p value
**Cerebral oxygen delivery in deep Gray Matter**			
Intercept	88	-654 – 832	0.82
Postmenstrual age, weeks	9	-8 – 26	0.32
Systemic-to-pulmonary shunt: yes	-22	-91 – 47	0.53
Aortic arch obstruction: yes	90	-14 – 194	0.09
**Cerebral oxygen delivery in cortical Gray Matter**			
Intercept	200	-298 – 698	0.44
Postmenstrual age, weeks	4	-8 – 16	0.51
Systemic-to-pulmonary shunt: yes	-33	-80 – 13	0.17
Aortic arch obstruction: yes	30	-39 – 98	0.41

Figure I. Differences in deep and cortical gray matter perfusion (A and B) and oxygen delivery (C and D) for patients with vs. without systemic-to-pulmonary shunt.

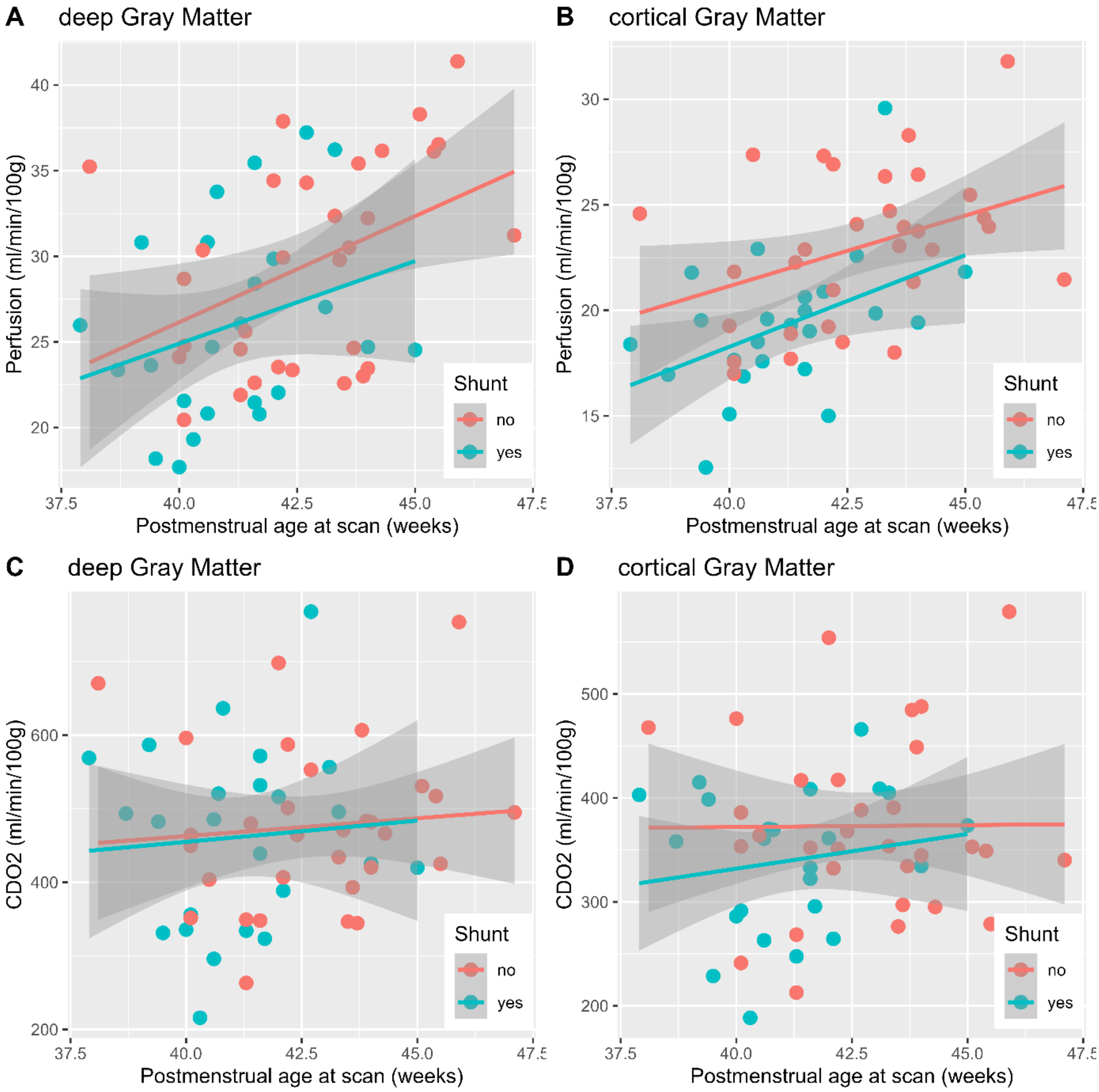

CDO_2: cerebral oxygen delivery

Note: Values are not adjusted for presence of aortic arch obstruction (perfusion and CDO_2 plots) and oxygen saturation (perfusion plots).
